# Supplementary material for: The effect of a multi-target protocol on cetacean detection and abundance estimation in aerial surveys
Source: R Soc Open Sci. 2019 Sep 4;6(9):190296. doi: 10.1098/rsos.190296 (PMC6774977; doi:10.1098/rsos.190296)
Supplement: Supplementary Files A [file rsos190296supp1.pdf]

Supplementary File A

Jags model for hierarchical modelling of the observer effect.

```
model{
  #####################
  ### meta analysis ###
  #####################

  ##############
  # parameters #
  ##############
  # xia, a, tau_a: random effect for observers
  # xib, b, tau_b: random effect for species
  # intercept: fixed effects
  # psi: occurrence proba

  ########
  # DATA #
  ########
  # n_obs, n_miss, n_observers, n_species
  # SPECIES
  # OBSERVERS
  # PROTOCOL
  # DISTANCE
  # DETECTED
  # PRESENT

  ##########
  # PRIORS #
  ##########

  # random effect with PX-Cholesky decomposition for observers
  for (j in 1:2) {
    A_a[j, j] ~ dnorm(0.0, 0.4444444)T(0.0,)
    Delta_a[j, j] <- 1/tau_a[j] ; tau_a[j] ~ dgamma(1.5, 1.5) ;
    L_a[j, j] <- 1.0;
  }
  L_a[1, 2] <- 0.0; A_a[1, 2] <- 0.0; Delta_a[1, 2] <- 0.0;
  L_a[2, 1] ~ dnorm(0.0, 4.0); A_a[2, 1] <- 0.0; Delta_a[2, 1] <- 0.0;
   # covariance matrix
  Omega_a <- A_a%*%L_a%*%Delta_a%*%t(L_a)%*%A_a;
   # random effects: bivariate normal
  for (k in 1:n_observers) {
    alpha[k, 1] <- A_a[1, 1]*(L_a[1, 1]*xia[k, 1]);
    alpha[k, 2] <- A_a[2, 2]*(L_a[2, 1]*xia[k, 1] + L_a[2, 2]*xia[k, 2]);
    for(j in 1:2){
      xia[k, j] ~ dnorm(0.0, tau_a[j]);
    }
  }
  sigma_alpha[1] <- sqrt(Omega_a[1, 1]); sigma_alpha[2] <- sqrt(Omega_a[2, 2]);
  rho_alpha[1] <- Omega_a[1, 2]/sqrt(Omega_a[1, 1]*Omega_a[2, 2]);

  # random effects with PX-Cholesky decomposition for species
  # covariance matrix
  Omega_b <- A_b%*%L_b%*%Delta_b%*%t(L_b)%*%A_b;
  sigma_beta[1] <- sqrt(Omega_b[1, 1]); sigma_beta[2] <- sqrt(Omega_b[2, 2]);
  rho_beta <- Omega_b[1, 2]/sqrt(Omega_b[1, 1]*Omega_b[2, 2]);
  for (l in 1:2) {
    A_b[l, l] ~ dnorm(0.0, 0.4444444)T(0.0,)
```

```
    Delta_b[l, l] <- 1/tau_b[l]; tau_b[l] ~ dgamma(1.5, 1.5);
    L_b[l, l] <- 1.0;
  }
  L_b[1, 2] <- 0.0; A_b[1, 2] <- 0.0; Delta_b[1, 2] <- 0.0;
  L_b[2, 1] ~ dnorm(0.0, 4.0); A_b[2, 1] <- 0.0; Delta_b[2, 1] <- 0.0;

  for(i in 1:n_species){
    beta[i, 1] <- A_b[1, 1]*(L_b[1, 1]*xib[i, 1]);
    beta[i, 2] <- A_b[2, 2]*(L_b[2, 1]*xib[i, 1] + L_b[2, 2]*xib[i, 2]);
    for(j in 1:2) {
      xib[i, j] ~ dnorm(0.0, tau_b[j]);
    }
  }

  # fixed effects
  for (l in 1:2) {
    intercept[l] ~ dnorm(0.0, 1.0);
  }
  psi ~ dunif(0.0, 1.0);

  ##############
  # likelihood #
  ##############
  for (j in 1:(n_obs+n_miss)){
    DISTANCE[j] ~ dunif(0.0, TRUNC);
    PRESENT[j] ~ dbern(psi);
    sigma[j] <- exp(intercept[PROTOCOL[j]] + beta[SPECIES[j], PROTOCOL[j]] + alpha[OBSERVER[j],
PROTOCOL[j]]);
    x[j] <- exp(-DISTANCE[j]*DISTANCE[j]/(2*sigma[j]*sigma[j]))*PRESENT[j];
    prob[j] <- max(0.0001, min(x[j], 0.9999)); # to avoid pb with initial values
    DETECTED[j] ~ dbern(prob[j]);
  }
}
```
