# Supplementary material for: The effect of a multi-target protocol on cetacean detection and abundance estimation in aerial surveys
Source: R Soc Open Sci. 2019 Sep 4;6(9):190296. doi: 10.1098/rsos.190296 (PMC6774977; doi:10.1098/rsos.190296)
Supplement: Supplementary Files B [file rsos190296supp2.pdf]

Supplementary File B

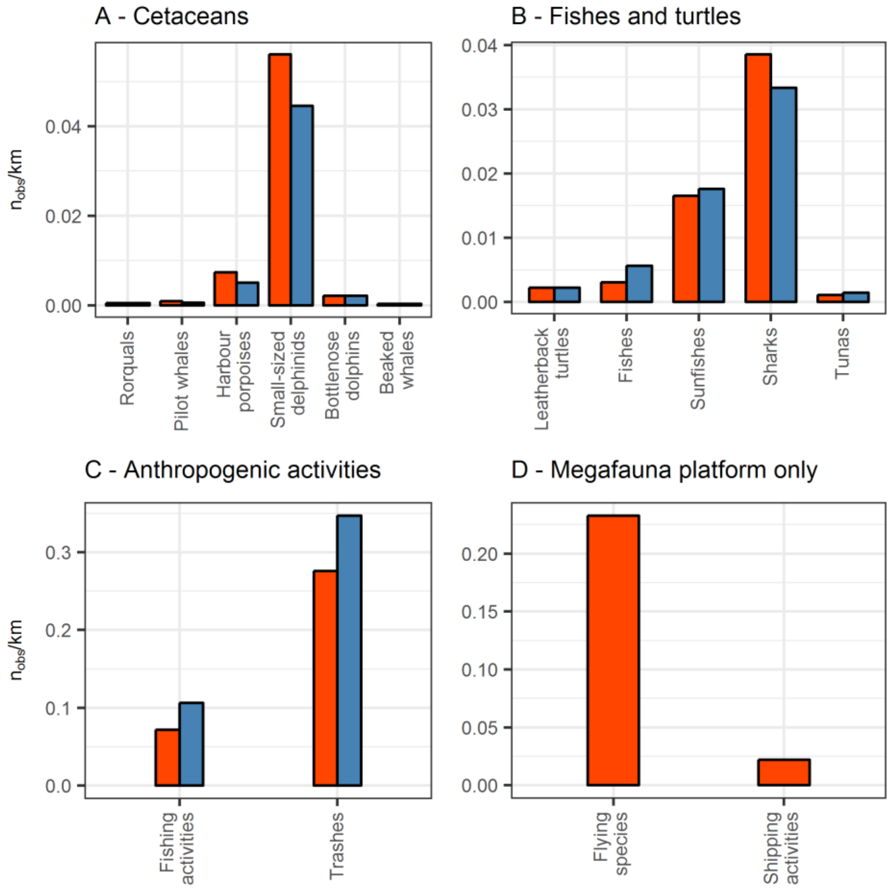

Figure 1. Encounter rates (Nobs/km) for (A) cetacean species groups, (B) fishes and turtles, (C) anthropogenic activities and (D) items recorded by the Megafauna platform only. Scans platform in blue, Megafauna platform in orange.

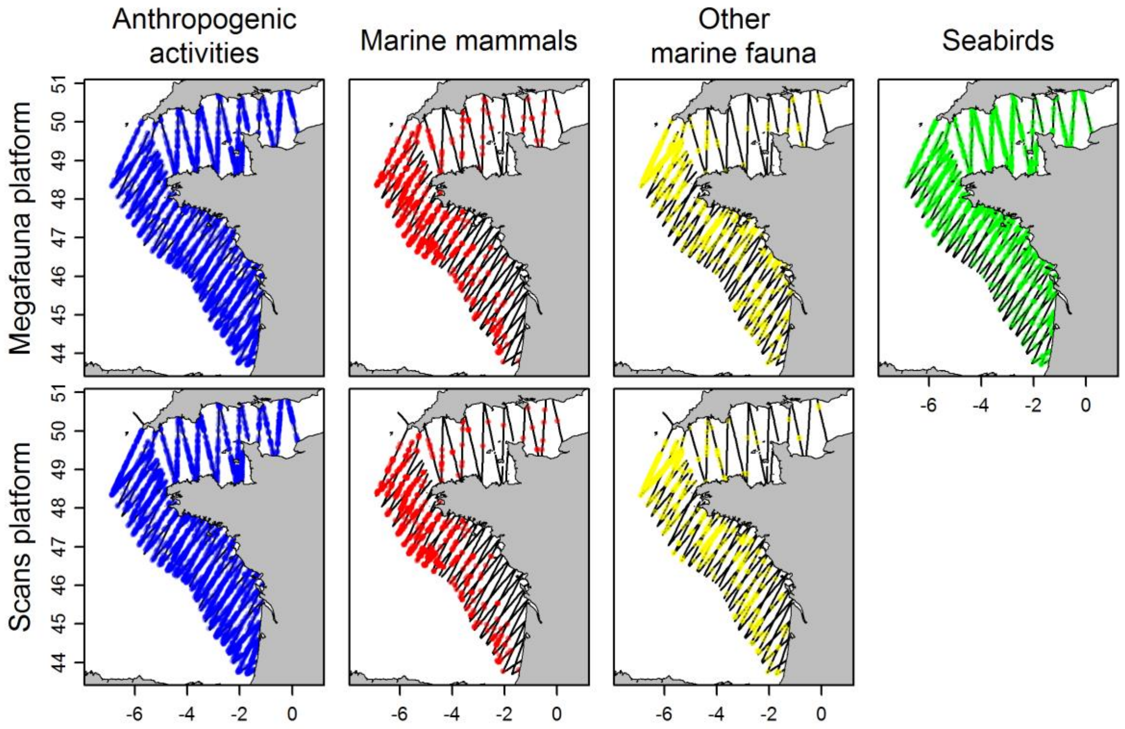

Figure 2. Sighting distributions for the four taxonomic groups encountered (marine mammals, other marine fauna, anthropogenic activities and seabirds), for Megafauna platform (above) and Scans platform (below).
