# Supplementary material for: The effect of a multi-target protocol on cetacean detection and abundance estimation in aerial surveys
Source: R Soc Open Sci. 2019 Sep 4;6(9):190296. doi: 10.1098/rsos.190296 (PMC6774977; doi:10.1098/rsos.190296)
Supplement: Supplementary Files C [file rsos190296supp3.pdf]

Supplementary File C

To test for the sensitivity of duplicate identifications (and subsequent perception probability estimation) to the assumptions of the decision tree, we manipulated the "Angle ± 15°" step.

Each tree step was verified manually when constructed, to check for the credibility of identified duplicates. The 500 m radius was chosen to take into account the uncertainty in sighting localisation inherent to protocol (due to the lag between actual detection by observer and actual recording by navigator, as well as due to the uncertainty associated to each platform independent GPS) and was thus not appropriate for the exercise.

The threshold of a 15° difference in detection angle was originally chosen from a manual exploration of the data, from the exploration of the difference in detection angle, and from expert knowledge (from the field perceptions of experienced observers).

A tighten and a looser decision tree were tested, corresponding to two different angle criterion: ±5 degree for the tighten tree and ±25 degree for the looser one.

Reminder: with the original decision tree, we identified 1047 unique sightings, among which 386 were duplicates. The estimated overall perception probability (from null model) was a bit higher for Megafauna platform (76%) than for Scans platform (65%).

Tighten decision tree (angle of ±5°)

The decision tree resulted in the identification of a set of 1205 unique sightings, among which 228 were recorded by both platforms (*i.e.*, were duplicates). The estimated overall perception probability (from null model) was a bit higher for Megafauna platform (61%) than for Scans platform (57%).

The difference in the perception probability *p* between the two platform in the absence of object detection was significant (*p*=0.54 for the Megafauna platform, *p*=0.46 for the Scans platform; Figure 3). Cetacean detection had a positive impact on *p* for both platforms, but was reduced compared to the original analysis (tree with angle ±15°). The other objects had no impact on the perception probability in both platforms.

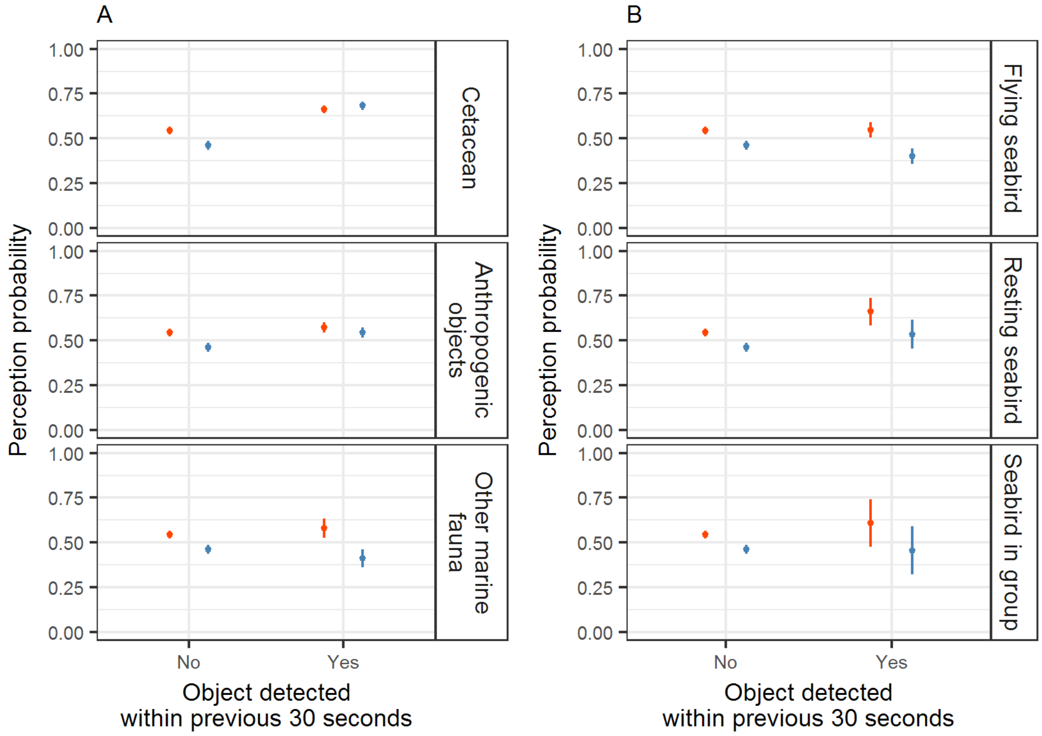

Figure 1. Small cetaceans perception probability estimated from the tighten tree by platform given the absence (No) or presence (Yes) of detection of other items during 30 seconds before the small cetacean sighting occurrences. A - effects of cetacean, other marine fauna and anthropogenic objects; B - effects of flying seabirds, resting seabirds and seabirds in groups. Megafauna platform values are in orange, Scans platform values in blue.

Looser decision tree (angle of ±25°)

The decision tree resulted in the identification of a set of 1022 unique sightings, among which 411 were recorded by both platforms (*i.e.*, were duplicates). The estimated overall perception probability (from null model) was a bit higher for Megafauna platform (73%) than for Scans platform (68%).

The difference in the perception probability *p* between the two platform in the absence of object detection was significant (*p*=0.64 for the Megafauna platform, *p*=0.55 for the Scans platform; Figure 5). Cetacean detection had a positive impact on *p* for both platforms, but was reduced compared to the original analysis (tree with angle ±15°). The other objects had no impact on the perception probability in both platforms.

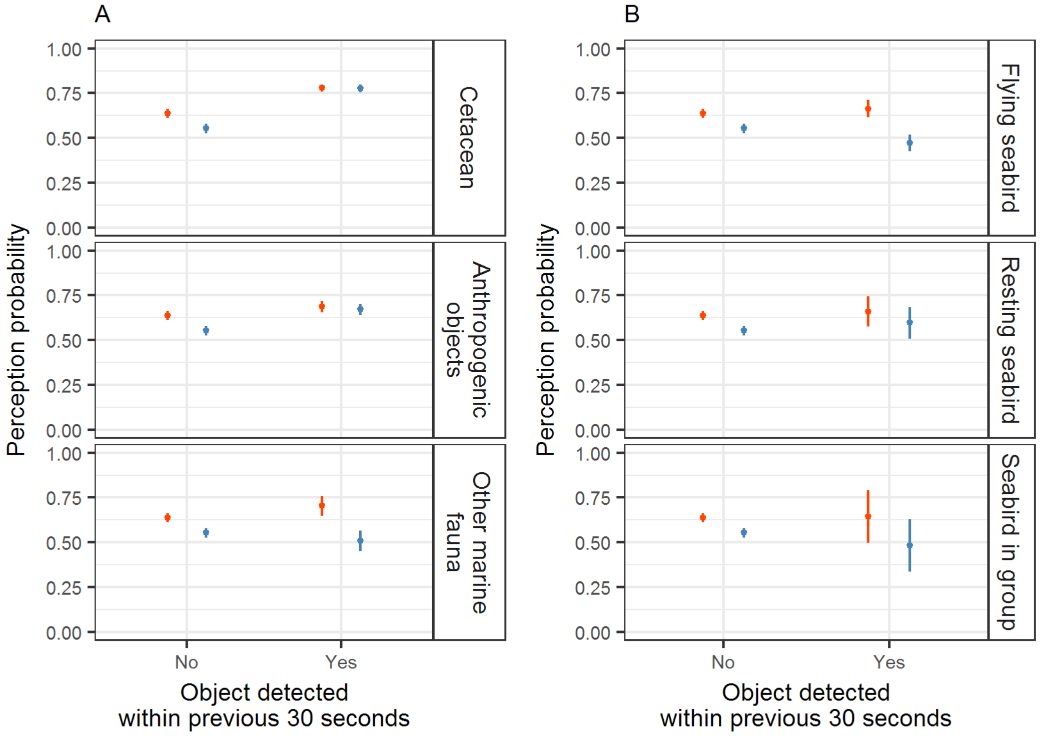

Figure 2. Small cetaceans perception probability estimated from the looser tree by platform given the absence (No) or presence (Yes) of detection of other items during 30 seconds before the small cetacean sighting occurrences. A - effects of cetacean, other marine fauna and anthropogenic objects; B - effects of flying seabirds, resting seabirds and seabirds in groups. Megafauna platform values are in orange, Scans platform values in blue.

Conclusion

The analysis performed here unveiled some sensitivity of our results to the decision tree:

- In all cases (5, 15, 25°), the perception probability of both platform (from null model) was estimated a bit higher for Megafauna platform
- In all cases (5, 15, 25°), the detection of a cetacean within the 30 seconds had a positive impact on the small cetacean perception probability
- In both cases (5, 25°), the effects of seabirds, other marine fauna and anthropogenic objects were lowered down (when not absent) compared to the 15° analysis

As a result, we can be confident in the main result from the perception probability estimation, *i.e.* that Megafauna platform do not perform poorly compared to the Scans platform, and that the cetacean detection increase the perception probability of small cetaceans. However, the patterns suggested from the original analysis (15°) on the effects of seabirds have to be interpreted with caution: these results provided some evidence that seabird detection lower the perception probability of small cetaceans, but analyses based on statistically more robust data are needed.
