# Supplementary material for: The effect of a multi-target protocol on cetacean detection and abundance estimation in aerial surveys
Source: R Soc Open Sci. 2019 Sep 4;6(9):190296. doi: 10.1098/rsos.190296 (PMC6774977; doi:10.1098/rsos.190296)
Supplement: Supplementary Files D [file rsos190296supp4.pdf]

Supplementary File D

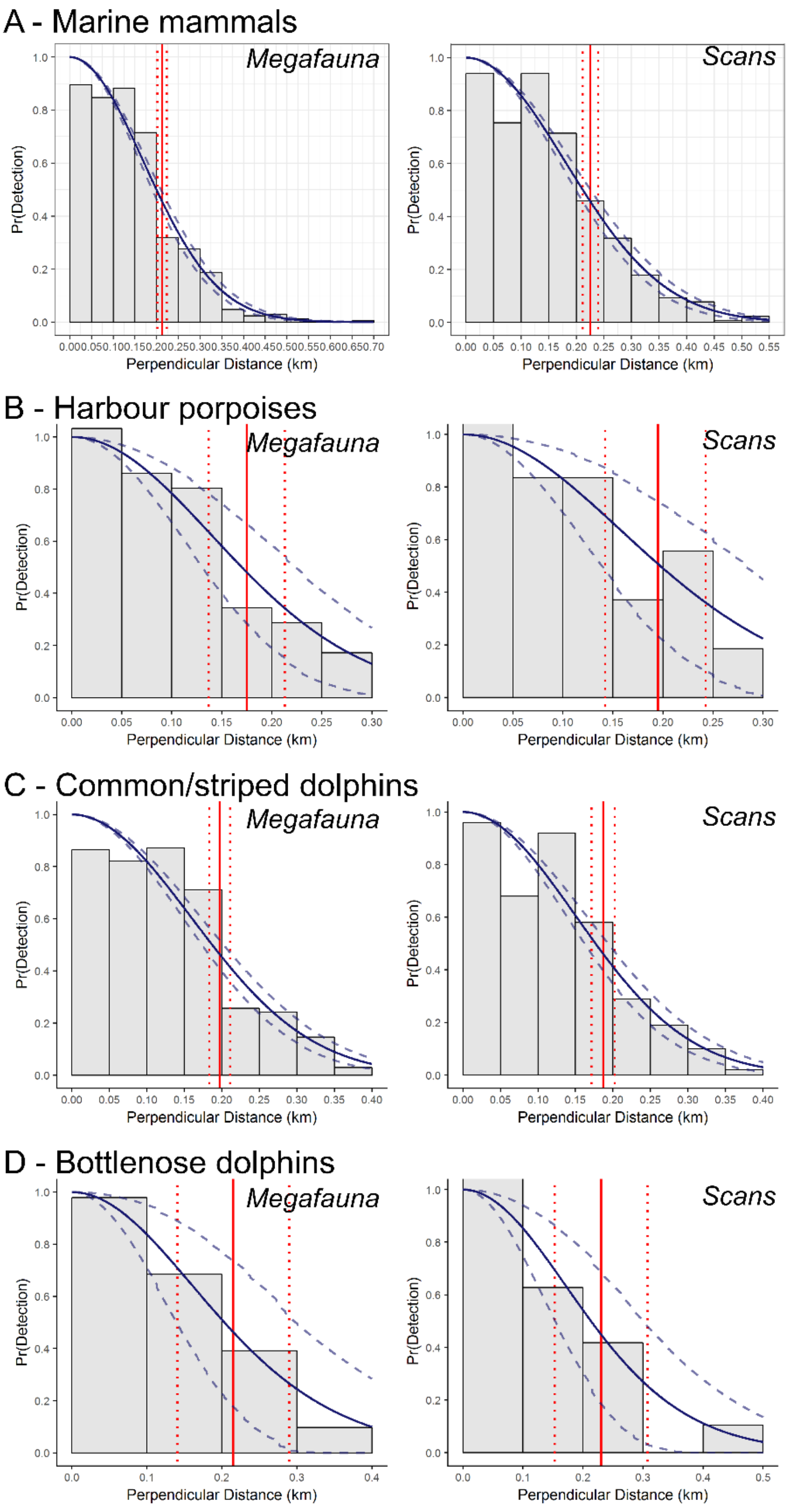

Figure 2. Detection functions fitted through distance sampling methodology for Megafauna (left) and Scans (right) platforms, for the four studied groups of cetaceans (A – marine mammals considered altogether; B – harbour porpoises; C – common/striped dolphins; D – bottlenose dolphins).

Table 1. CDS results by platform for harbour porpoise (A), common/striped dolphins (B) and bottlenose dolphin (C). The CV of encounter rates, group size, abundance and density estimates are provided in parenthesis. CDS was done over sightings made in good observation conditions (Beaufort sea state < 4 and moderate to excellent subjective conditions) for harbour porpoises and common/striped dolphins; all sightings were used for bottlenose dolphins. Half-normal key was used for the detection functions of the three groups.

Platform	Sector	Truncation distance (km)	N obs	N ind	Estimated encounter rate (N(ind)/km)	Estimated pod size (individuals)	Abundance (individuals)	Density (ind/km)	ESW (km)
**A - Harbour porpoises**									
Megafauna	Bay of Biscay	0.28	17	42	0.006 (0.343)	2.471 (0.218)	2080 (0.360)	0.018 (0.360)	0.175 [0.137 ; 0.213]
	English Channel		44	84	0.044 (0.247)	1.909 (0.151)	10252 (0.269)	0.126 (0.269)	
	Total		61	126	0.014 (0.203)	1.985 (0.130)	12332 (0.240)	0.062 (0.240)	
Scans	Bay of Biscay	0.29	10	24	0.004 (0.495)	2.400 (0.356)	1231 (0.513)	0.01 (0.513)	0.192 [0.141 ; 0.240]
	English Channel		33	66	0.038 (0.275)	2 (0.158)	8015 (0.307)	0.099 (0.307)	
	Total		43	90	0.011 (0.242)	2.045 (0.144)	9247 (0.282)	0.046 (0.282)	
**B - Small-sized delphinids**									
Megafauna	Bay of Biscay	0.4	537	3096	0.447 (0.125)	5.765 (0.071)	134706 (0.130)	1.137 (0.130)	0.197 [0.183 ; 0.211]
	English Channel		1	15	0.008 (0.998)	15 (0)	1608 (0.999)	0.020 (0.999)	
	Total		538	3111	0.352 (0.125)	5.808 (0.070)	136314 (0.129)	0.682 (0.129)	
Scans	Bay of Biscay	0.4	372	2834	0.472 (0.155)	7.618 (0.117)	149848 (0.160)	1.265 (0.160)	0.187 [0.171 ; 0.202]
	English Channel		2	24	0.014 (0.848)	12 (0.473)	3002 (0.849)	0.037 (0.849)	
	Total		374	2858	0.369 (0.155)	7.673 (0.115)	152850 (0.158)	0.765 (0.158)	
**C - Bottlenose dolphins**									
Megafauna	Bay of Biscay	0.34	22	171	0.021 (0.337)	7.773 (0.194)	5988 (0.378)	0.051 (0.378)	0.215 [0.141 ; 0.290]
	English Channel		0	0	0	0	0	0	
	Total		22	171	0.016 (0.338)	7.773 (0.194)	5988 (0.378)	0.030 (0.378)	
Scans	Bay of Biscay	0.411	22	158	0.020 (0.302)	7.182 (0.175)	5156 (0.343)	0.044 (0.343)	0.230 [0.153 ; 0.308]
	English Channel		0	0	0	0	0	0	
	Total		22	158	0.015 (0.302)	7.182 (0.175)	5156 (0.343)	0.026 (0.343)	

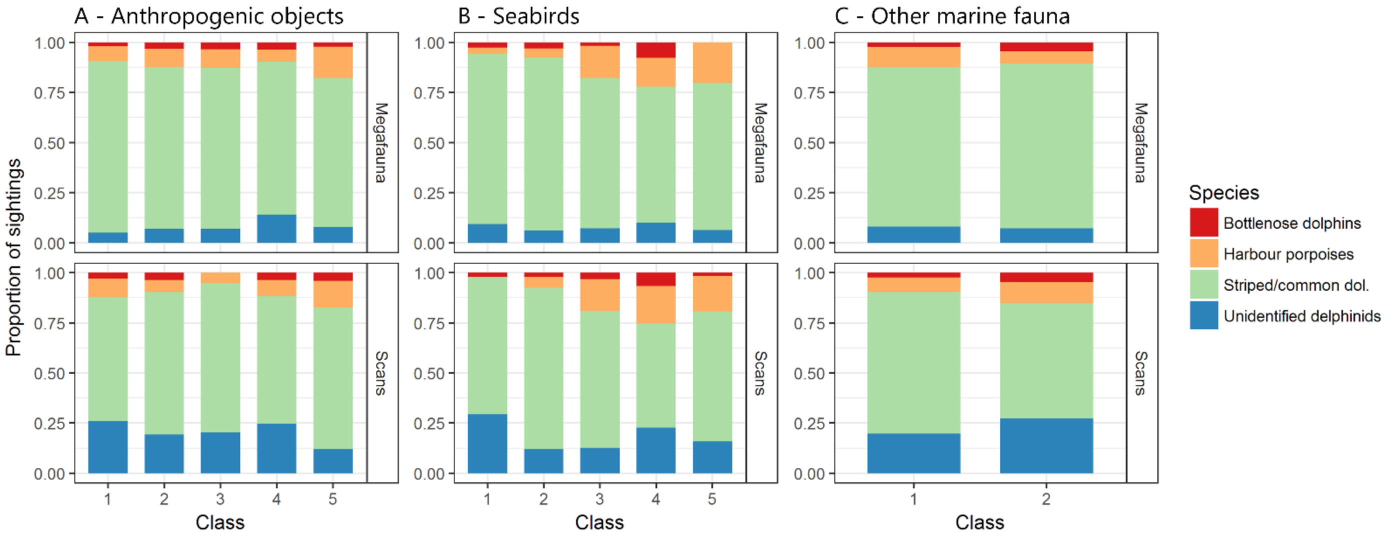

Figure 3. Cetacean species composition for each post-stratified class of anthropogenic objects (A), seabirds (B) and other marine fauna (C), for Megafauna (above) and Scans (below) platforms.

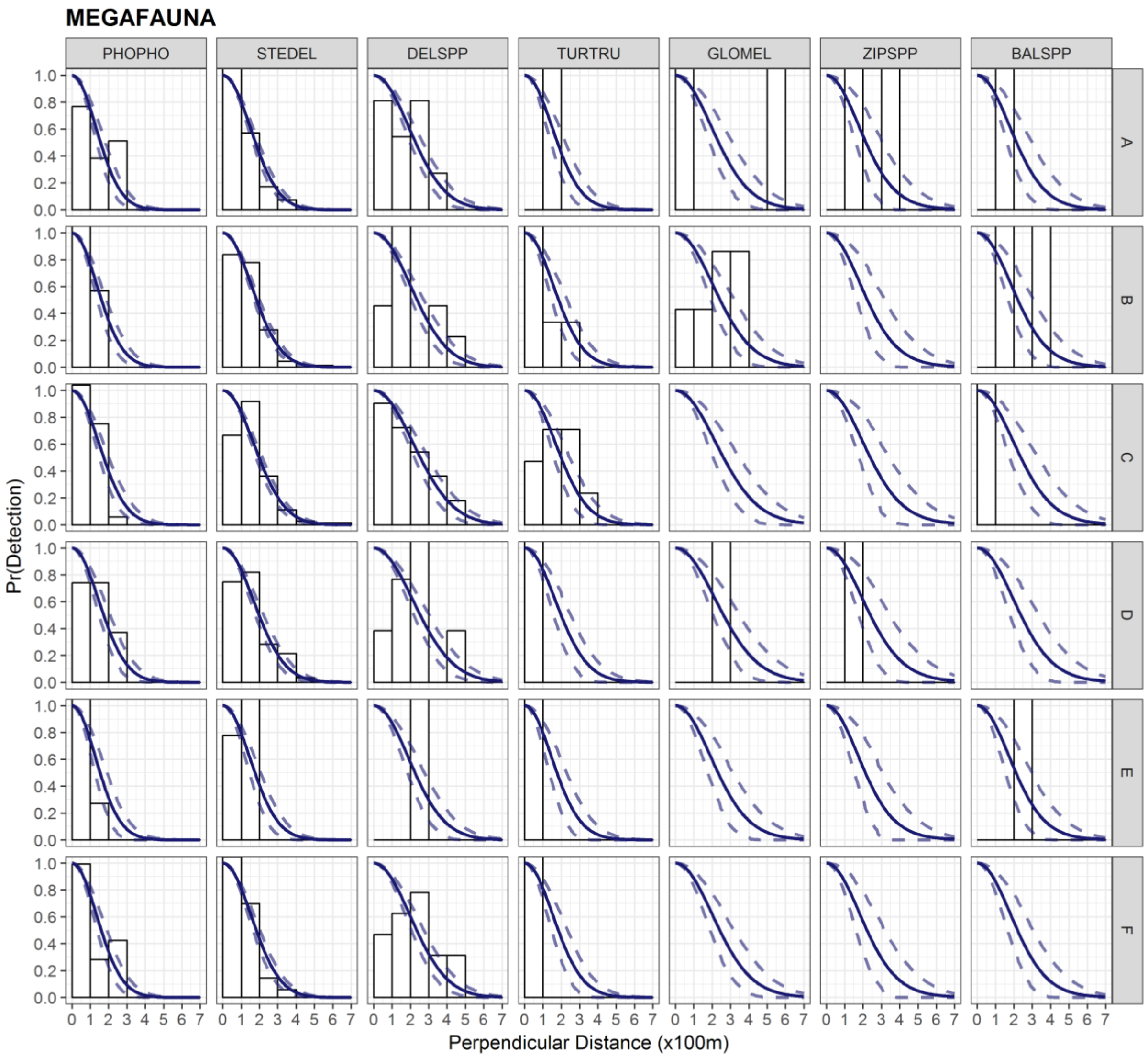

Figure 4. Distance histograms (in white), averaged detection functions (in blue) and associated uncertainty (in dashed blue) for each considered group of species and by observer (A-F), for the Megafauna platform.

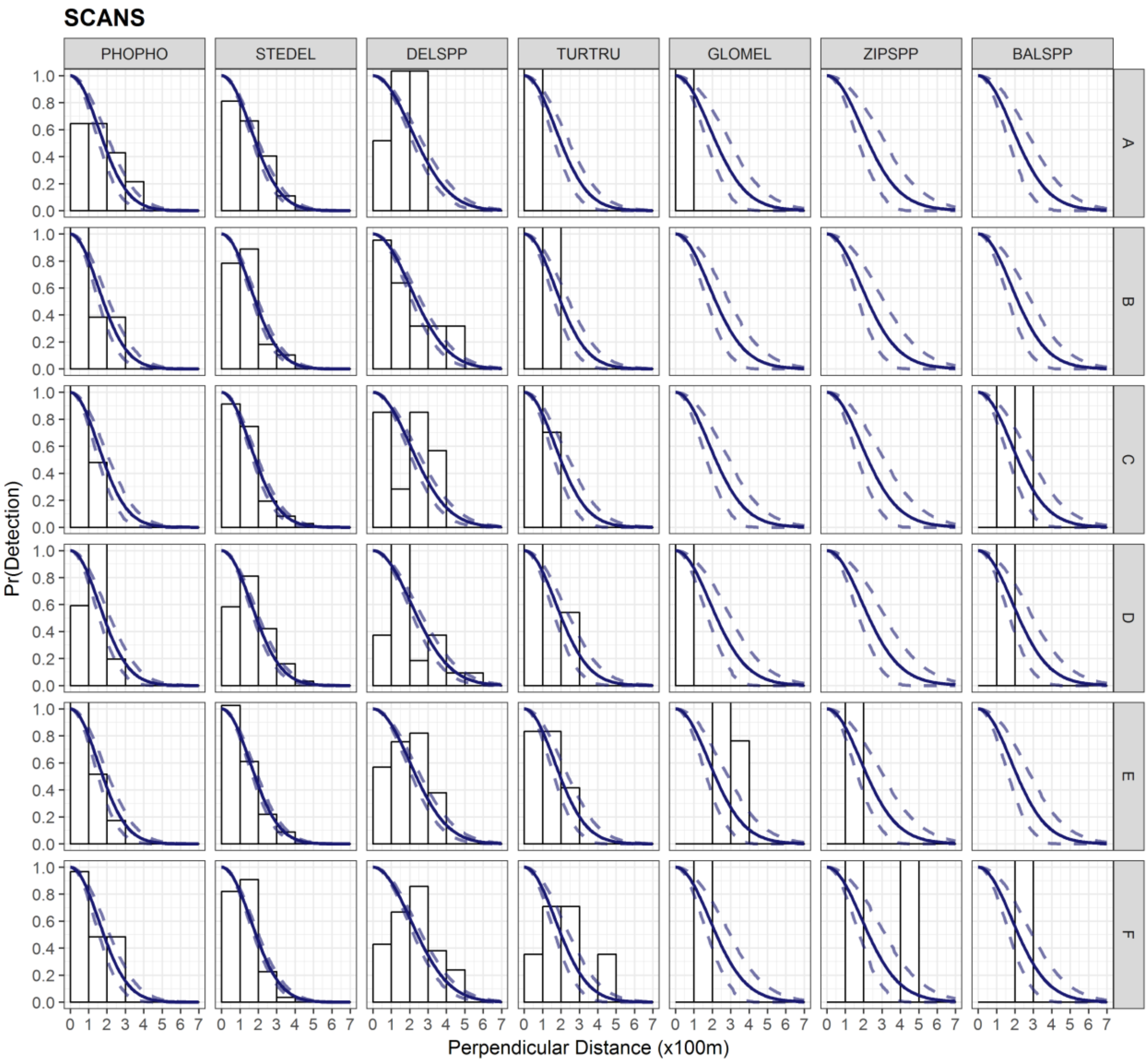

Figure 4. Distance histograms (in white), averaged detection functions (in blue) and associated uncertainty (in dashed blue) for each considered group of species and by observer (A-F), for the Megafauna platform
