# Supplementary material for: The effect of a multi-target protocol on cetacean detection and abundance estimation in aerial surveys
Source: R Soc Open Sci. 2019 Sep 4;6(9):190296. doi: 10.1098/rsos.190296 (PMC6774977; doi:10.1098/rsos.190296)
Supplement: Supplementary Files E [file rsos190296supp5.pdf]

Supplementary File E

One Size Fits All: Multi-species Aerial Surveys May Not Be Biased Compared to Single Species Surveys

Matthieu Authier[1,2], Sophie Laran[1], Dorémus Ghislain[1], Olivier Van Canneyt[1], and Vincent Ridoux[1]

[1] Observatoire PELAGIS, UMS-CNRS 3462, Université de la Rochelle, Pôle Analytique, allée de l'Océan, 17000 La Rochelle, FRANCE

[2] mauthier@univ-lr.fr

One Size Fits All: Multi-species Aerial Surveys May Not Be Biased Compared to Single Species Surveys

Matthieu Authier[1,2], Sophie Laran[1], Dorémus Ghislain[1], Olivier Van Canneyt[1], and Vincent Ridoux[1]

[1]Observatoire PELAGIS, UMS-CNRS 3462, Université de la Rochelle, Pôle Analytique, allée de l'Océan, 17000 La Rochelle, FRANCE

[2]mauthier@univ-lr.fr

June 20, 2016

Aims

This short note details the results of a prospective power analysis to detect a difference between two protocols. The SCANS protocol targets harbour porpoises (*Phocoena phocoena*) but does not record other marine megafauna (seabirds, large fish, *etc*). The Megafauna protocol on the other hand targets all megafauna species visible from the air. The question is to assess whether the Megafauna protocol can deliver data on harbour porpoises of commensurable quality with that collected with the SCANS protocol. To answer this question, a prospective power analysis was carried out. Moreover, a preliminary analysis of a pilot experiment with the simultaneous implementation of the two protocols (hereafter referred to as miniSAMM) by independent teams was undertaken. The two main aims were to:

1. assess detectable magnitude of a bias in small cetacean detection of the Megafauna protocol compared to the SCANS protocol; and to

2. assess the relative magnitude of observer effects compared to protocol effects from a pilot study.

Power Study

Data Simulation

Based on a previous pilot study (miniSAMM), we can reasonably expect to obtain between 150 and 250 detections of Harbour Porpoises for the allocated effort for the "Double Plateform" experiment. We conducted data simulations for expected sample size between 150 and 250 by increment of 25. It can be anticipated that statistical power will depend on this sample size, with larger sample size leveraging more power to detect smaller bias.

Data simulation were carried out with a negative bias for the Megafauna protocol. The bias on detection could manifest itself in two different ways (see Figure 1).

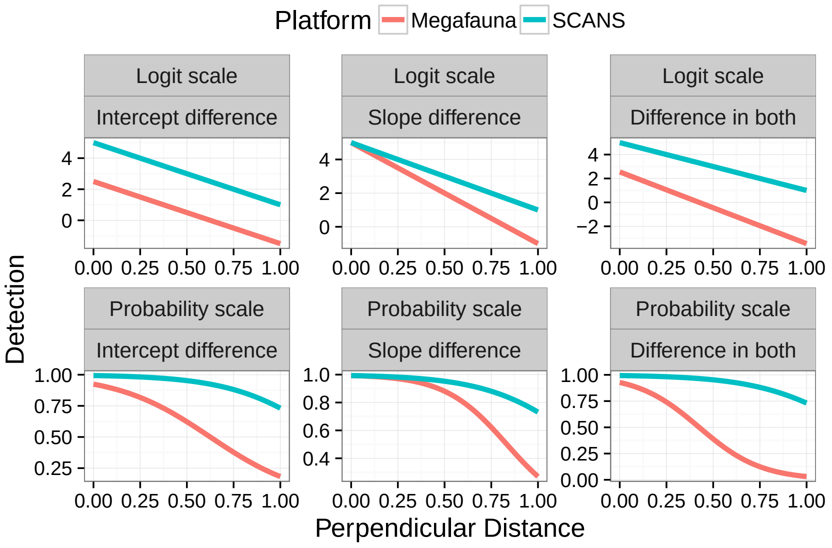

Figure 1: Examples of an assumed relationship between the probability of detecting an animal, and the distance of the said animal to the transect line. The assumed relationship is linear on a logit scale (top row), but non-linear on the probability scale (bottom row). Leftmost panel: intercept difference in the detection pattern. Middle panel: slope difference in the detection pattern. Rightmost panel: intercept and slope differences in the detection pattern.

For given animal present in the survey area, it can either be detected ($\mathrm{D} = 1$) or not ($\mathrm{D} = 0$). Let $\mathrm{D}^{\mathrm{scan}}$ and $\mathrm{D}^{\mathrm{mega}}$ denote detection by the SCANS and Megafauna platforms respectively. For the same animal, the possible observed capture histories $\mathrm{D}^{\mathrm{scan}}\mathrm{D}^{\mathrm{mega}}$ are 11 if the animal is detected by both platforms, 10 if it is detected only by the SCANS platform, 01 if it is detected only by the Megafauna platform. The capture history 00 is never observed.

Detections were modelled as Bernoulli trials:

$$\begin{cases} \mathrm{D}^{\mathrm{scan}} \sim \mathrm{Bernoulli(p)}, \ \mathrm{logit(p)} = \beta_0^{\mathrm{scan}} + \beta_1^{\mathrm{scan}} \times \mathrm{PerpDist} \\ \mathrm{D}^{\mathrm{mega}} \sim \mathrm{Bernoulli(q)}, \ \mathrm{logit(q)} = \beta_0^{\mathrm{mega}} + \beta_1^{\mathrm{mega}} \times \mathrm{PerpDist} \end{cases} \tag{1}$$

A difference between the two protocols can manifest itself in the intercept (β_0) or the slope (β_1). We considered only a negative bias for the megafauna protocol. In the simplest case, the probability to detect on the line ($\mathrm{PerpDist} = 0$) was lower:

$$\mathrm{logit}^{-1}\left(\beta_0^{\mathrm{mega}}\right) = \delta_0 \times \mathrm{logit}^{-1}\left(\beta_0^{\mathrm{scan}}\right) \tag{2}$$

where $0 < \delta_0 < 1$ is the bias.

We also considered a difference between slopes:

$$\beta_1^{\mathrm{mega}} = (1 + \delta_1) \times \beta_1^{\mathrm{scan}} \tag{3}$$

where $0 < \delta_1 < 1$ is the bias. Since the sign of the slope is negative, this results in $\beta_1^{\mathrm{mega}} < \beta_0^{\mathrm{mega}}$, that is a shorter effective strip width for the megafauna platform compared to the SCANS one.

Reasonable values for the parameters $(\beta_0^{\mathrm{scan}}, \beta_1^{\mathrm{scan}})$ were obtained from a pilot study carried in late winter 2013 in the Channel, in which a double platform experiment was carried out for 14 hours of flight effort ($2,450$ km). A total of 150 detections of harbour porpoises were realized. Values for $\mathrm{logit}^{-1}\left(\beta_0^{\mathrm{scan}}\right)$ ranged between 0.3 and 0.9 by increment of 0.1. Values for β_1^{scan} ranged between -1.5 and -0.5 by increment of 0.2.

Values for the bias parameters (δ_0, δ_1) ranged between 0.05 and 0.50 by increment of 0.05.

For each combination of these parameter values, 500 datasets were simulated in `R` (R Development Core Team, 2015) version 3.2.3. These datasets were then analyzed with the package `unmarked` (Fiske & Chandler, 2011) to estimate the magnitude of a bias that was detectable with 80% statistical power at the 5% risk level.

Results

For all scenarios envisioned here, a bias in the slope parameter δ_1 was not detectable: in other words, even with a the sample size as large as 250 and a bias as large as 50%, statistical power was lower than 80% to detect a difference in slope. This result is unsurprising given the non-linear transform which maps the logit scale to the probability scale (Figure 1): a difference in the intercept on the logit scale can change the slope of the relationship on the probability scale (compare the leftmost and rightmost panels of Figure 1) [1].

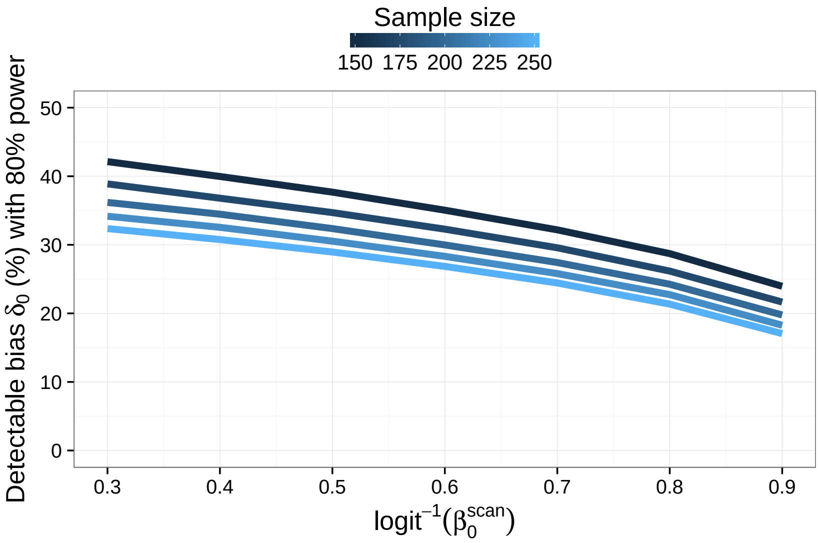

Figure 2

The magnitude of a detectable bias in the detection probability on the transect line for the megafauna platform depended on the detection probability on the transect line for the SCANS platform: the higher this latter probability, the smaller the detectable bias (Figure 2). Unsurprisingly, the larger the sample size, the smaller the detectable bias. However, even in the most favorable scenario (sample size of 250 and 0.9 detection probability on the transect line for the SCANS platform), the smallest detectable bias was already larger than 15%. Thus, in this configuration, if the detection on the transect line for the megafauna platform is smaller than $0.9 \times (1 - 0.15) \approx 0.77$, it would be detected at the 5% risk level with 80% power.

[1] This interaction without an explicit statistical interaction term in the model is due to a phenomena called "compression", which results from the mapping of an unrestricted latent variable on the logit scale to the restricted $[0, 1]$ probability interval (Berry et al., 2010).

Values obtained from the pilot study, were $\text{logit}^{-1}\left(\beta_0^{\hat{\text{scan}}}\right) = 0.6$ and $\beta_1^{\hat{\text{scan}}} = -1.0$. Thus, with a sample size of 150 detections, a bias of 35% was detectable with 80% statistical power. In other words, detection on the transect line for the megafaune platform must be smaller than $0.6 \times (1 - 0.35) \approx 0.4$ to be statistically detectable.

However, a more surprising result from this pilot survey was that detection was actually better with the megafauna protocol (Figure 3).

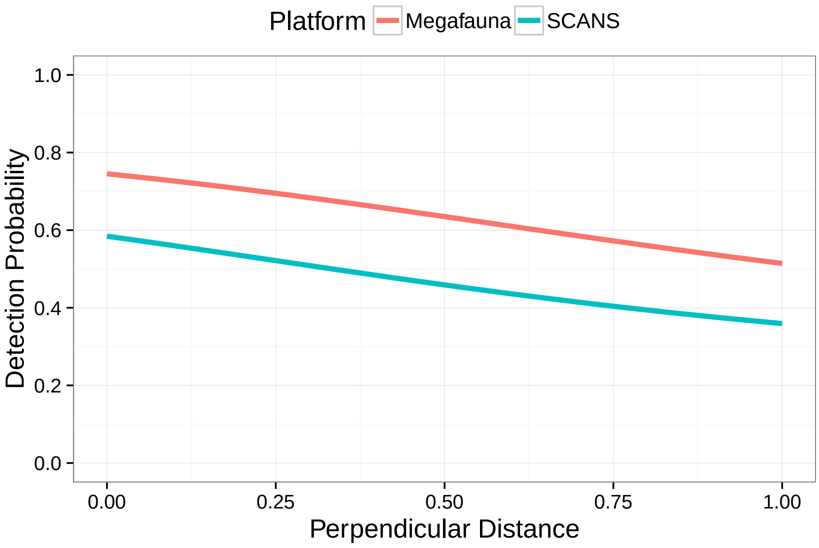

Figure 3

Observer Effects

Another important bias beyond that of the protocol is that of observers. We had a brief look at observer bias, again with the miniSAMM pilot study. Specifically, we fitted the following random effects model to the data:

$$\begin{cases} \mathrm{D^{scan}}\sim\mathrm{Bernoulli(p)},\ \mathrm{logit(p)}=\beta_0^{\mathrm{scan}}+\beta_1^{\mathrm{scan}}\times\mathrm{PerpDist}+\alpha_{\mathrm{i}}^{\mathrm{scan}} \\ \mathrm{D^{mega}}\sim\mathrm{Bernoulli(q)},\ \mathrm{logit(q)}=\beta_0^{\mathrm{mega}}+\beta_1^{\mathrm{mega}}\times\mathrm{PerpDist}+\alpha_{\mathrm{i}}^{\mathrm{mega}} \end{cases} \tag{4}$$

where $(\alpha^{\mathrm{scan}}, \alpha^{\mathrm{mega}})_i$ are observer effects. Observer effects were larger in magnitude than the estimated effect between the two protocols (Figure 4). In fact, in the miniSAMM study, observers were nested within a protocol, and thus any protocol effect was confounded with observer effects. On Figure 4, the protocol effect is actually 0 while the previously identified difference (Figure 3) is visible as an observer effect. While the miniSAMM design prevents the joint estimation of an observer and protocol effects, the miniSAMM data nevertheless suggest that any difference between the two protocols may be dwarfed by between-observers variability.

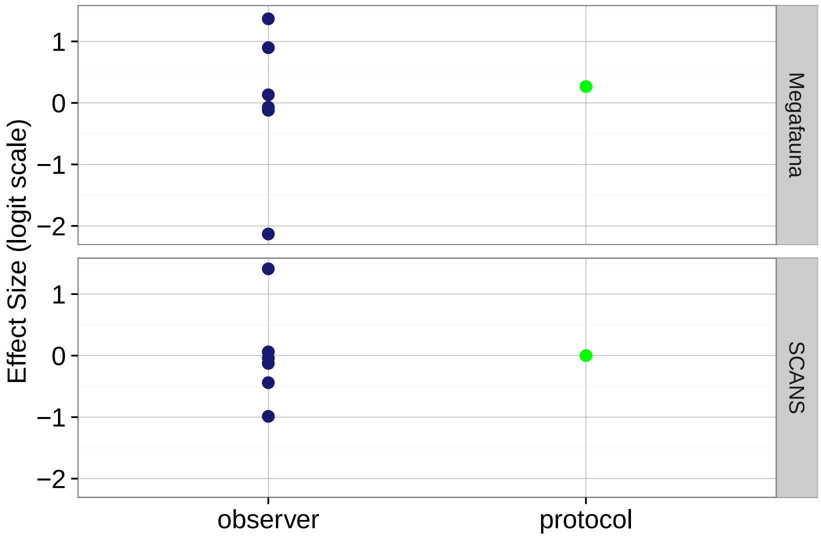

Figure 4

Acknowledgments

We are grateful to Andrew Trites for constructive comments of a draft of this short note. We thank all the observers of the miniSAMM survey.

References

BERRY, D. B., DEMERITT, J. H. R. & ESAREY, J. (2010). Testing for Interaction in Binary Logit and Probit Models: Is a Product Term Essential? *American Journal of Political Science* 54 248–266.

FISKE, I. & CHANDLER, R. (2011). `unmarked`: an `R` Package for Fitting Hierarchical Models of Wildlife Occurrence and Abundance. *Journal of Statistical Software* 43 1–23. URL `http://www.jstatsoft.org/v43/i10/`.

R DEVELOPMENT CORE TEAM (2015). *R: A Language and Environment for Statistical Computing*. R Foundation for Statistical Computing, Vienna, Austria, r version 3.2.3 (2015-12-10) – "wooden christmas-tree" ed. ISBN 3-900051-07-0, URL `http://www.R-project.org/`.
